# Antibacterial, antifungal, and antibiofilm activities of biogenic zinc nanoparticles against pathogenic microorganisms

**DOI:** 10.3389/fcimb.2025.1545119

**Published:** 2025-07-14

**Authors:** Eliana Daniela Lopez Venditti, Karina Fernanda Crespo Andrada, Pamela Soledad Bustos, Manuela Maldonado Torales, Iván Manrrique Hughes, María Gabriela Paraje, Natalia Guiñazú

**Affiliations:** ^1^ Centro de Investigaciones en Toxicología Ambiental y Agrobiotecnología del Comahue (CITAAC), Consejo Nacional de Investigaciones Científicas y Técnicas (CONICET), Neuquén, Argentina; ^2^ Departamento de Ciencias del Ambiente y la Salud, Facultad de Ciencias del Ambiente y la Salud, Universidad Nacional del Comahue, Neuquén, Argentina; ^3^ Cátedra de Microbiología, Facultad de Ciencias Exactas, Físicas y Naturales, Universidad Nacional de Córdoba, Córdoba, Argentina; ^4^ Instituto Multidisciplinario de Biología Vegetal (IMBIV), Consejo Nacional de Investigaciones Científicas y Técnicas (CONICET), Córdoba, Argentina; ^5^ Departamento de Ciencias Farmacéuticas, Facultad de Ciencias Químicas, Universidad Nacional de Córdoba, Haya de la Torre y Medina Allende, Córdoba, Argentina

**Keywords:** biogenic zinc nanoparticles, antibacterial activity, antifungal activity, biofilm associated infections, oxidative stress, nitrosative stress, total antioxidant capacity

## Abstract

**Introduction:**

The increasing resistance to antimicrobial drugs has prompted global efforts to combat pathogenic bacteria and fungi. The World Health Organization’s recent report underscores the urgent need for innovative antimicrobial strategies to address infections caused by *Staphylococcus aureus*, *Escherichia coli*, *Candida albicans*, and *Candida tropicalis*. This study presents a comparative evaluation of the effects of biogenically synthesized zinc nanoparticles (ZnNPs) from *Pseudomonas aeruginosa*, highlighting their effectiveness against both planktonic and sessile forms of these tested pathogens.

**Methods:**

The antimicrobial effects were assessed using the Kirby-Bauer disk diffusion method, broth microdilution, and time-kill assays. Biofilm formation and eradication were evaluated through crystal violet staining, resazurin assays, and colony-forming unit quantification. Additionally, the oxidative and nitrosative stress toxicity mechanisms triggered by ZnNPs, particularly those related to cellular stress, were investigated.

**Results:**

The results demonstrated that ZnNPs exhibit concentration-dependent inhibitory effects on both prokaryotic and eukaryotic microorganisms. ZnNPs inhibit biofilm formation by up to 50% in *E. coli* and yeast species, and up to 80% in *S. aureus*.

**Discussion:**

These antibiofilm activities were attributed to disruptions in cellular stress metabolism, primarily driven by nitrosative stress through enhanced production of reactive nitrogen intermediates. ZnNPs synthesized through green methods offer significant advantages due to their biocompatibility and potential biomedical applications. These findings advance our understanding of ZnNPs in combating biofilm-associated infections, offering promising strategies to address pathogenic bacteria and fungi, which pose a critical threat to global health.

## 1 Introduction

The increasing resistance to antimicrobial drugs has complicated the effective treatment of infections, leading to the establishment of global priorities and guidelines to improve the management of pathogenic bacteria and fungi, which pose a critical threat to global health. The World Health Organization (WHO) recently published a report prioritizing pathogenic bacteria and fungi to guide research and public health efforts in combating resistant infections. This report highlights key bacteria and fungi associated with antimicrobial resistance and underscores the urgent need for innovative strategies, including the development of new antimicrobial agents (Vitiello et al., 2023; WHO, 2024). For instance, *Staphylococcus aureus*, particularly methicillin-resistant *S. aureus*, is classified as a high priority due to its widespread resistance and significant burden on healthcare systems. Similarly, *Escherichia coli*, especially extended-spectrum beta-lactamase (ESBL)-producing and carbapenem-resistant strains, is listed as a critical priority because of its association with life-threatening infections and limited therapeutic options. Among fungal pathogens, *Candida albicans* is recognized as a critical priority, while *Candida tropicalis* is classified as a high priority (Jesudason, 2024).

Biogenic metal nanoparticles (NPs) have significant potential in biomedical applications, providing innovative solutions to various health challenges. In particular, microbial resistance to conventional antimicrobial treatments and the persistent infections caused by bacteria and fungi represent critical health concerns that can potentially be addressed using metal NPs (Paraje, 2023; Arora et al., 2024). Both bacteria and yeast have evolved various virulence strategies to ensure infection persistence, one of the most significant being biofilm formation. Biofilms are structured, three-dimensional communities of microorganisms that play a crucial role in infection development and persistence, significantly increasing antimicrobial resistance compared to their planktonic (free-floating) counterparts (Paraje, 2023; Coenye et al., 2024).

Zinc (Zn), a trace metal essential for human health, is critical for optimal enzyme activity across various organs and systems. It plays a fundamental role in numerous physiological and metabolic processes (Costa et al., 2023). Zn is a promising candidate for NPs production due to its high reduction potential. As an active element and a powerful reducing agent, Zn can be easily oxidized to form zinc oxide (ZnO) (Kalaba et al., 2024). Zinc nanoparticles (ZnNPs) and zinc oxide nanoparticles (ZnO NPs) have emerged as promising materials for various medical applications due to their biocompatibility, antimicrobial activity, and multifunctional properties (Król et al., 2017). This property makes Zn advantageous for the synthesis of ZnO NPs, which have potential applications for nano-optical and nano-electronic devices, as well as in food packaging and medicine, where they exhibit antimicrobial and antitumor properties (Costa et al., 2023; Masoudi et al., 2024).

Biological methods have been developed for NP synthesis and various studies have demonstrated that bacteria can produce metal NPs both intra- and extracellularly (Kulkarni et al., 2023; Rasheed et al., 2024). Intracellular biosynthesis occurs within the bacterial biomass, whereas extracellular biosynthesis is achieved using cell-free extracts or bacterial culture supernatants (Borehalli Mayegowda et al., 2023). Particularly, *E. coli* and *Pseudomonas aeruginosa* have been shown to produce metallic NPs from iron, silver, and gold salts. Among these, *P. aeruginosa* can transform metal salts into stable metallic NPs through biochemical processes involving enzymes, proteins, and polysaccharides that act as reducing and stabilizing agents. This environmentally friendly and cost-effective green synthesis approach, combined with *P. aeruginosa*’s adaptability to diverse environmental conditions and rapid growth rate, makes it a promising candidate for the sustainable biosynthesis of ZnNPs (Crespo et al., 2016; Ihsan et al., 2023; Okaiyeto et al., 2024).

This research hypothesizes that biogenic ZnNPs display antimicrobial and antibiofilm capabilities, in both bacteria and yeast strains. Thus, the present study explored the antimicrobial and antibiofilm activities of ZnNPs synthesized with green methods, obtained by microbial synthesis using *P. aeruginosa*. For this purpose, a comparative evaluation was conducted to assess the antimicrobial activity against both planktonic and sessile cells of two bacterial strains, *S. aureus* and *E. coli*, as well as two yeast strains, *C. albicans* and *C. tropicalis*. Research also focused on ZnNP toxicity mechanisms by the determination of reactive oxygen species (ROS), reactive nitrogen intermediates (RNI) and total oxidative stress response (OSR), in the bacteria and yeast treated with the ZnNPs.

To the best of our knowledge, this is the first study to comprehensively investigate the multifunctional antimicrobial of biogenic ZnNPs synthesized by *P. aeruginosa*, demonstrating their effectiveness against both bacterial and yeast biofilms. Our findings provide valuable insights into how these eco-friendly nanoparticles combat biofilm-associated infections caused by *S. aureus, E. coli, C. albicans*, and *C. tropicalis*. Furthermore, the development of novel strategies to eradicate biofilms holds significant clinical promise, particularly for treating the growing population of at-risk patients.

## 2 Materials and methods

### 2.1 Reagents

Trypticase Soy Broth (TSB), Trypticase Soy Agar (TSA), Sabouraud Dextrose Broth (SDB), and Sabouraud Dextrose Agar (SDA) were purchased from Difco (MI, USA) and prepared according to the manufacturer’s instructions. Muller-Hinton Broth (MHB) and Mueller-Hinton agar (MHA) were obtained from Britania (Buenos Aires, Argentina) and prepared as per the manufacturer’s guidelines. Zinc sulfate heptahydrate (ZnSO_4_·7H_2_O), NaNO_2_, FeSO_4_, FeCl_3_·6H_2_O and Crystal Violet (CV) were acquired from Cicarelli (Santa Fe, Argentina). Resazurin-resorufin (Alamar Blue, AB), Amphotericin B (AmB), Nitro Blue Tetrazolium (NBT), fetal bovine serum sulfanilamide, N-1-naphthyl ethylene diamine dihydrochloride, Roswell Park Memorial Institute (RPMI) 1640 medium, D-glucose, glutamine, morpholinepropanesulfonic acid, and 2,4,6-tripyridyl-s-triazine, were sourced from Sigma-Aldrich Co. (St. Louis, MO, USA). Ciprofloxacin (CIP) was supplied by Roemmers (Buenos Aires, Argentina). HCl, dimethyl sulfoxide (DMSO), ethanol, acetic acid, and glycerol were obtained from Anedra (Buenos Aires, Argentina). Phosphate-buffered saline (PBS) 10x stock (8% NaCl, 0.2% KCl, 1.44% Na_2_HPO_4_, 0.24% KH_2_PO_4_) was prepared and filter-sterilized. Petri dishes and flat-bottom 96-well plates were provided by Greiner Bio-One (Frickenhausen, Germany).

### 2.2 Bacterial and fungal strains and biogenic ZnNPs

The bacterial strains *S. aureus* ATCC 29213 and *E. coli* ATCC 25922 were used for antimicrobial studies (Arce Miranda et al., 2011; Angel Villegas et al., 2015). Additionally, the yeast strains *C, albicans* SC 5314 and *C. tropicalis* NCPF 311 were employed (Miranda et al., 2019; Da Silva et al., 2021). All strains were stored in cryovials containing 20% glycerol at −80°C until use in the *in vitro* studies. For antimicrobial activity assays, the bacterial genus was subcultured on TSA, while the yeast genus was subcultured on SDA. The cultures were incubated at 37°C for 24 hours prior to use. The biogenic ZnNPs described in previous studies were utilized in this work (Crespo et al., 2016; Okaiyeto et al., 2024). Experimental flowchart including biosynthesis and following experiments is shown as Supplementary material 1 (**Supplementary Figure S1**).

The ZnNPs physicochemical properties are detailed in **Supplementary Material 2**, which includes UV-Vis spectroscopy (**Supplementary Figure S2A**), zeta potential analysis (**Supplementary Figure S2B**), transmission electron microscopy (TEM; **Supplementary Figure S2C**), and scanning electron microscopy (SEM; **Supplementary Figure S2D**). Additionally, **Supplementary Material 3** presents Fourier-transform infrared (FT-IR) spectroscopy (**Supplementary Figure S3A**), SDS-PAGE with silver staining (**Supplementary Figure S3B**), and fluorescence (FL) spectroscopy (**Supplementary Figure S3C**). Together, these analyses provide comprehensive information on the size, surface charge, morphology, and surface chemistry of the ZnNPs.

### 2.3 Determination of microbial susceptibility to ZnNPs by the Kirby-Bauer method

MHB was used to prepare the agar medium for bacterial cultures. Fresh overnight cultures of *S. aureus* and *E. coli* were grown in TSB until reaching the logarithmic growth phase. The bacterial suspensions were standardized by adjusting the turbidity to 0.5 McFarland standards with McFarland Densitometer DEN-1B (Britania, Argentina). For the candidal species, fresh overnight cultures of *C. albicans* and *C. tropicalis* were prepared in SDB and standardized to 0.5 McFarland (Kourmouli et al., 2018).

The inoculum was uniformly swabbed onto individual Petri dishes containing MHA, following the Clinical and Laboratory Standards Institute (CLSI) guidelines (Clinical and Laboratory Standards Institute, 2018). A sterile cotton swab, moistened with each inoculum suspension, was used to inoculate 30 mL of MHA in 90-mm diameter plates. The agar plates were left to dry for 3–15 minutes, after which wells were created using a cork borer (5 mm diameter). Reference antibiotics agents (CIP and AmB, in DMSO 1% v/v), biogenic ZnNPs, biosynthesis supernatant (SN) and ZnSO_4_, were dispensed into the wells on the inoculated plates, which were subsequently incubated at 37°C for 24 hours. After incubation, diameter of the zones of inhibition was measured using a standard caliper (mm), based on three independent determinations performed in duplicate. The zone of clearing around each well was measured to evaluate the susceptibility of the tested microorganisms, taking into account the diameter of the wells themselves (5 mm ± 1 mm).

The percentage inhibition of diameter growth (PIDG) was calculated to evaluate the antimicrobial activity of the ZnNPs relative to the positive control. PIDG values were estimated according to the following equation (Himratul-Aznita et al., 2011):


$$PIDG\left(\%\right)=\frac{Diameter\;of\;trated\;sample-Diameter\;of\;positive\;control}{Diameter\;of\;positive\;control}X\;100$$

### 2.4 Antibacterial and antifungal activity in planktonic cells

An important limitation of the Kirby-Bauer method is that it does not provide a minimum inhibitory concentration (MIC) value. The MIC was defined as the lowest concentration of the antimicrobial agent that resulted in a 99.9% reduction of the initial inoculum after subculturing onto fresh media. Therefore, the MIC, the minimum bactericidal concentration (MBC) and the minimum fungicidal concentration (MFC) were determined using the standard tube dilution method, following the guidelines established by the CLSI (Clinical and Laboratory Standards Institute, 2017, 2020). The antibacterial and antifungal effects of ZnNPs were assessed using overnight cultures of each microorganism, diluted to achieve a cell density equivalent to 0.5 on the McFarland scale. Serial dilutions of ZnNPs (ranging from 0 to 6600 µg/mL) were prepared in a 96-well microtiter plate and incubated at 37°C for 24 hours in triplicate (Pierce et al., 2008; Da Silva et al., 2022, 2024; Herman et al., 2024).

To assess metabolic activity and confirm the MIC value, the resazurin-resorufin (AB) assay was employed, based on the redox status of microbial cells (Herman et al., 2024). An aliquot of 20 µL of a 0.02% (w/v) AB solution was added to each well and incubated at 37°C for 30 minutes. Following incubation, the absorbance was recorded at 570 nm using a microplate reader (Infinite F50 Model, Tecan, AUS). Color changes in the wells were evaluated: pink indicated microbial cell viability, while blue signified microbial cell death. The AB solution was aliquoted and stored at −80°C to maintain stability and was brought to room temperature before use (Da Silva et al., 2022, 2024).

For MBC and MFC determination, viable cell counts were obtained from samples that showed no visible growth after incubation at 37°C for 24 hours and the colony-forming units (CFU) were counted and expressed as CFU/mL (Peralta et al., 2015, 2016, 2018; Da Silva et al., 2022, 2024). The lowest concentration that prevented the growth of bacterial or yeast colonies on antifungal- or antibiotic-free media was defined as the MBC or MFC, respectively. Each assay was performed in triplicate, and the mean percentage reduction in metabolic activity was used to confirm the MIC.

Controls included growth controls, SN and sterility controls. Strain viability and medium sterility were monitored concurrently to ensure the reliability of the results. Antibiotic or antifungal solution (CIP and AmB) were used as positive controls, dissolved in DMSO (1% v/v) to enhance solubility and stability. Activities were classified as bactericidal (MBC/MIC ≤ 4) or bacteriostatic (MBC/MIC > 4), and as fungicidal (MFC/MIC ≤ 4) or fungistatic (MFC/MIC > 4) based on these ratios (Arce Miranda et al., 2011; Angel Villegas et al., 2015; Da Silva et al., 2022, 2024).

### 2.5 Time-kill curves

A time-kill curve assay was performed using at MIC and higher concentrations and cell densities for each sample were determined by performing colony counts. This involved diluting the samples in PBS (pH 7.2 ± 0.1), plating them on TSA Petri dishes for bacteria and SDA for *Candida* species, and enumerating colony-forming units (CFU) to estimate viable cell counts, with a detection limit of 100 CFU/mL. To ensure reliability and reproducibility, each assay was performed in triplicate. Positive controls included CIP for *E. coli* and *S. aureus* and AmB for *C. albicans* and *C. tropicalis* were included. A negative control, referred to as the growth control, was also included, where microbial cells were incubated without ZnNPs for the same duration (Da Silva et al., 2021; Yakoup et al., 2024).

### 2.6 Antimicrobials susceptibility assay in biofilms

Based on the method described by O’Toole and Kolter (1998), mature biofilm formation was quantified using CV staining in 96-well polystyrene microtiter plates (O’Toole and Kolter, 1998; Peralta et al., 2015, 2016, 2018; Da Silva et al., 2021, 2022, 2024). Biofilms of each studied species were prepared by adding 100 µL of a suspension standardized to 1.0 McFarland in each well of flat-bottomed 96-well microtiter plates. To allow mature biofilm formation, after a 90-minute cell adhesion period, the wells were gently washed three times with sterile PBS (pH 7.2 ± 0.1). Then, TSB (200 µL) was added to each well, and the plates were incubated at 37°C for 24 hours (Peralta et al., 2015, 2016, 2018; Da Silva et al., 2021, 2022, 2024). Different concentrations of ZnNPs, including sub-MIC level (1/2 MIC), the MIC, and supra-MIC levels (5×, 10×, 25×, 50× and 100× MIC), were added to wells containing mature biofilms. The samples were then incubated at 37°C for 24 hours. After the incubation time, the supernatant was collected for ROS, RNI, and antioxidants quantification assays, and the wells were rinsed three times with PBS and air-dried for 24 hours (Peralta et al., 2015, 2016, 2018; Da Silva et al., 2021, 2022, 2024).

Biofilms were stained with 200 µL of 1% (w/v) CV solution per well for 5 minutes. After staining, the CV solution was removed, and the wells were washed three times with 300 µL of PBS pH 7.2 ± 0.1. For the quantification of mature biofilm, CV was extracted with 200 µL of an ethanol/glacial acetone solution for 5 minutes (70:30, v/v).

Absorbance was measured spectrophotometrically at 595 nm using a microplate reader (Infinite F50 Model, Tecan, AUS). The biofilm biomass unit (BBU) was arbitrarily defined as 1 BBU = 0.1 optical density (OD)_595nm_. Untreated biofilms were considered positive controls for mature biofilm formation, and the OD of the control wells was subtracted from that of all tested wells. Each strain was tested in three independent experiments conducted on different days to ensure reproducibility (Peralta et al., 2016; Da Silva et al., 2021, 2022).

Sessile Minimum Inhibitory Concentration (SMIC50 and SMIC80) for ZnNPs was determined based on a 50% and 80% reduction in absorbance of mature biofilm biomass following treatment with the NPs, compared to untreated control biofilms formed under identical conditions.

The percentage of biofilm reduction was calculated using the following equation (Arce Miranda et al., 2011; Angel Villegas et al., 2015; Peralta et al., 2015, 2016, 2018; Da Silva et al., 2021, 2022, 2024):


$$\;Reduction\;\left(\%\right)=\frac{BBU\;untreated\;-BBU\;treated\;}{BBU\;untreated}\;x\;100$$

### 2.7 Minimal biofilm eradication concentration

The minimal biofilm eradication concentration (MBEC) was determined by the cultivable sessile cells of mature biofilms after exposure to the ZnNPs using plate counting (CFU/mL), 24 hours of incubation at 37°C. The MBEC50 represents a 50% reduction in viable sessile cells, while the MBEC80 represents an 80% reduction in viable sessile cells of mature biofilm. To resuspend and homogenize the sessile cells, the microplate was sonicated for 1 minute at 40 kHz. Subsequently, 100 μL of sterile PBS previously diluted in serial dilution in PBS were seeded onto a TSA or SDA plate and incubated for 24-48 h at 37°C. The MBEC determined by the AB assay was defined as the reduction of mature biofilm biomass by ≤50% or 80% following the addition of AB. The sessile “Live cells” correspond to the measurement obtained from AB staining, and “Total biofilm” is determined based on the biomass (sessile cells and extracellular matrix) measurement from CV staining (Peralta et al., 2015, 2016, 2018; Da Silva et al., 2021, 2022, 2024; Herman et al., 2024):

The percentage of viability was calculated using the following equation:


$$\;Viability\;\left(\%\right)=\frac{AB\;or\;CFU\;\left(Live\;Cells\right)}{BBU\;\left(Live\;Cells+Dead\;Cells+EPS\right)}\;x\;100$$

### 2.8 Quantification of oxidative and nitrosative stress

ROS production was quantified spectrophotometrically (Baronetti et al., 2011; Arce Miranda et al., 2021). In brief, 100 µL of supernatant separated was incubated with 100 µL of NBT for 30 minutes at 37°C, and the absorbance was measured at 540 nm using a microplate reader (Arce Miranda et al., 2011, 2021; Baronetti et al., 2011; Angel Villegas et al., 2015; Peralta et al., 2018, 2015, 2016; Miranda et al., 2019; Quinteros et al., 2021b, 2021a; Da Silva et al., 2021, 2022, 2024).

The production of RNI was assessed indirectly using the Griess reaction, which quantifies its stable degradation products, nitrate and nitrite. For this assay, 50 µL of supernatant was mixed with 200 µL of Griess reagent, consisting of sulfanilamide (1.5% in 1 N HCl) and N-1-naphthyl ethylene diaminedihydrochloride (0.13% in sterile distilled water). Absorbance was recorded at 540 nm. Sodium nitrite (1 mM) was used as the standard, and a calibration curve was constructed in the range of 100–0.05 mM (Arce Miranda et al., 2011; Baronetti et al., 2013; Angel Villegas et al., 2015; Da Silva et al., 2021; Haddi et al., 2024).

### 2.9 Total oxidative stress response

The determination of the OSR, enzymatic and non-enzymatic systems, was assessed using the Ferric Reducing Antioxidant Power (FRAP) assay. Microbial suspensions were incubated with 2,4,6-tripyridyl-1,3,5-triazine (TPTZ) in 40 mM HCl, 0.125 mL of 5.4 mg/mL FeCl_3_Æ6H_2_O plus 1.25 mL of 300 mM acetate buffer (pH 3.6). The production of ferrous tripyridyltriazine (Fe²^+^-TPTZ) complex was evaluated at 595 nm. The FRAP values were calculated using a FeSO_4_ calibration curve (Arce Miranda et al., 2011; Angel Villegas et al., 2015; Peralta et al., 2015, 2016, 2018; Da Silva et al., 2021, 2022, 2024; Quinteros et al., 2021b, 2021a).

### 2.10 Statistical analysis

Statistical analysis was conducted using triplicate groups, and each experiment was repeated three times (n = 9). The data are presented as means ± standard deviation. ANOVA was used for analysis, followed by the Student-Newman-Keuls test for multiple comparisons, with *p < 0.1 and ** p < 0.01 for differences compared to untreated biofilms. ^#^p <0.01 differences considered significant for comparisons of ZnNPs and the antimicrobial controls (CIP and AmB) or SMIC and MBEC. Graphical statistical analysis was carried out using GraphPad Prism 6.0 and Microsoft Excel.

## 3 Results

### 3.1 Susceptibility of planktonic bacteria and yeast to ZnNPs

**Figure 1:**
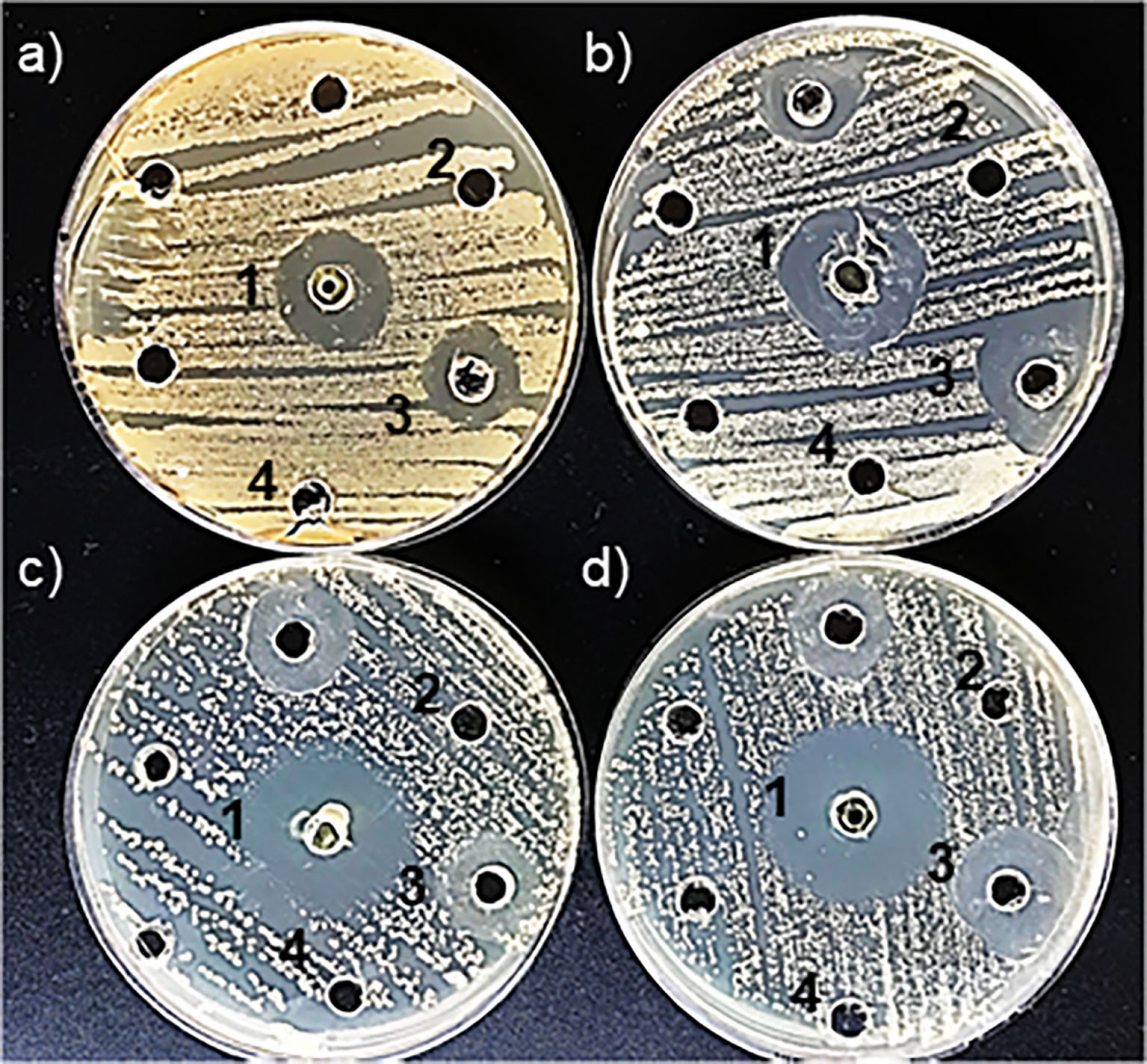
Detection of antimicrobial susceptibility using the Kirby-Bauer disk diffusion method by ZnNPs. **(a)** Inhibition halos produced for *E coli*, **(b)** *S. aureus*, **(c)** *C. albicans*, and **(d)** *C. tropicalis*. (1) Positive control: CIP for *E coli* and *S. aureus*; AmB for *C. albicans* and *C tropicalis*. (2) ZnNPs; (3) SN; (4) ZnSO_4_. Each assay was conducted with three independent determinations, each performed in duplicate.

The disk diffusion method is one of the oldest techniques used to evaluate the antimicrobial activity of novel compounds. This method assesses the efficacy of a compound by measuring the diameter of the inhibition zone, which reflects its antimicrobial susceptibility against the tested microorganism (Kourmouli et al., 2018). In this study, the antimicrobial activity of biogenic ZnNPs was evaluated using the Kirby-Bauer disk diffusion method against planktonic bacterial and yeast cells, specifically *E. coli* ATCC 25922, *S. aureus* ATCC 29213, *C. albicans* SC 5314, and *C. tropicalis* NCPF 3111. All tested microorganisms exhibited inhibition halos, confirming the antimicrobial activity of ZnNPs. **Figures 1A–D** illustrate the inhibition zones (halos), while **Table 1** provides quantitative data on inhibition diameters in millimeters (mm), ranging from -18.76 ± 1.00 mm to 28.74 ± 1.74 mm. Wells containing CIP served as positive controls for bacterial species, while AmB was used for yeast. Negative controls, including SN and ZnSO_4_, showed no inhibition halos. The ZnNPs exhibited significant antibacterial activity, with inhibition zone diameters of 28.74 ± 1.74 mm for *S. aureus* and 21.04 ± 0.34 mm for *E. coli*. In contrast, the antifungal activity was lower, as indicated by the inhibition zone of -18.76 mm for *C. albicans* and 26.110 ± 0.86 for *C. tropicalis*.

**Table 1:** Inhibition zone diameters and PIDG of ZnNPs against different pathogens.

Strains	Inhibition zone diameters (mm)				PIDG (%)
	Positive control	ZnNPs	ZnSO_4_	SN	
*E. coli* ATCC 25922	25.90 ± 0.55	21.04 ± 0.34	–	–	-18.76
*S. aureus* ATCC 29213	32.94 ± 0.24	28.74 ± 1.74	–	–	-12.75
*C. albicans* SC 5314	39.90 ± 1.13	19.88 ± 1.00	–	–	-50.18
*C. tropicalis* NCPF 3111	41.20 ± 0.90	26.10 ± 0.86	–	–	-36.65

Percentage inhibition of diameter growth (PIDG), millimeters (mm), biogenic ZnNPs, biosynthesis supernatant (SN).

The PIDG (%) was calculated to assess the antimicrobial activity of the ZnNPs in comparison to the positive control. Positive control: Ciprofloxacin (for *E. coli* and *S. aureus*) and Amphotericin B (for *C. albicans* and *C. tropicalis*); (-) indicates the absence of inhibition zones. All data were the means obtained from three sets of tests carried out in duplicates.

Based on the PIDG values, which reflect the strength of antimicrobial activity in relation to established antimicrobial agents used in treatments. All tested bacteria and yeasts were found to be susceptible to the biogenic ZnNPs. Higher PIDG values correspond to greater antimicrobial activity. Higher PIDG values correspond to greater antimicrobial activity. However, the negative PIDG values observed indicate that the antimicrobial activity of the ZnNPs was generally weaker than that of the positive control. In this study, the positive control was the reference antimicrobial drug, while the negative control represented the baseline with no antimicrobial effect. Therefore, negative PIDG values mean that ZnNPs showed lower antimicrobial activity compared to the reference drug but still exhibited inhibitory effect. The antimicrobial activity of ZnNPs was comparatively weaker than that of the antimicrobial control, as indicated by negative PIDG values. A comparative analysis demonstrated that planktonic bacterial cells were generally more susceptible to ZnNPs than planktonic yeast cells, with *S. aureus* exhibiting the highest susceptibility (-12.75) and *C. albicans* the lowest (-50.18) (**Table 1**).

The MIC, MBC and MFC were determined using the standard micro broth dilution method on planktonic bacteria and yeast cells treated with varying concentrations of ZnNPs (Clinical and Laboratory Standards Institute, 2017, 2020). The use of AB facilitated the visualization of MIC values for both bacterial and fungal strains (Herman et al., 2024). **Table 2** presents the quantitative antibacterial and antifungal activity data. The MIC for *S. aureus* and *E. coli* was determined to be 200 µg/mL and 100 µg/mL, respectively, while the MBC for both bacterial strains was 200 µg/mL.

**Table 2:** Quantitative data from MIC and MBC assays of ZnNPs against planktonic bacterial and fungal cells.

Strains	Planktonic cells			
	MIC	MBC	MBC/MIC	INTPN
*S. aureus* ATCC 29213	200	200	1	Bactericide
*E. coli* ATCC 25922	100	200	2	Bactericide
	MIC	MFC	MFC/MIC	INTPN
*C. albicans* SC 5314	200	400	2	Fungicide
*C. tropicalis* NCPF 3111	200	200	1	Fungicide

MIC, minimum inhibitory concentration; MBC, minimum bactericidal concentration; MFC, minimum fungicidal concentration; INTPN, interpretation.

All MIC and MFC data are expressed in terms of µg/mL for ZnNPs. Ciprofloxacin: *E. coli* ATCC 25922: MIC 0.012 µg/mL, MBC 0.12 µg/mL; and *S. aureus* ATCC 29213: MIC 0.25 µg/mL, MBC 2 µg/mL. Amphotericin B: *C. albicans* SC 5314: MIC and MFC 0.25 µg/mL; and *C. tropicalis* NCPF 311: MIC and MFC 0.5 µg/mL.

The MIC and MFC values for planktonic yeast cells were determined to evaluate their susceptibility to ZnNPs. Both *C. albicans* and *C. tropicalis* reached fungicidal endpoints at 200 µg/mL, which is within the normal range for susceptible strains. However, the MFC for *C. albicans* was 400 µg/mL, whereas for *C. tropicalis*, it was 200 µg/mL.

Additionally, the MBC/MIC and MFC/MIC ratios were calculated to determine the microbicidal efficacy of the ZnNPs. The final column of **Table 2** provides the interpretation (INTPN) of the correlation between MIC and MBC values for bacteria, as well as MIC and MFC values for yeast strains. ZnNPs demonstrated effective bactericidal and fungicidal activity, with a ratio ≤ 4.

### 3.2 Time-kill assay for evaluating antimicrobial activity of ZnNPs

**Figure 2:**
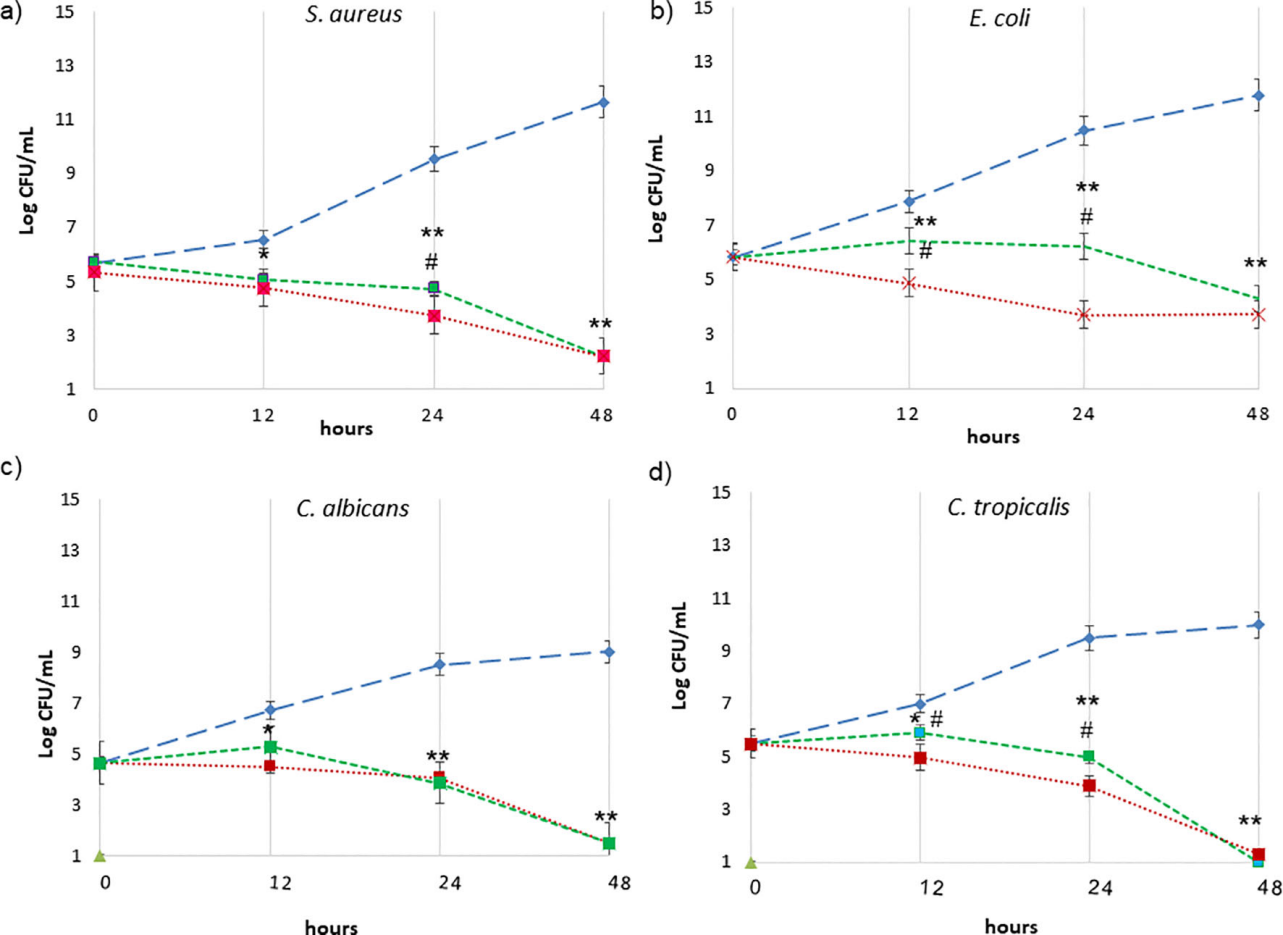
Time-kill curves expressed as log_10_ CFU/mL versus incubation time. ZnNPs at the MIC concentrations are represented by the green lines as follows: **(a)** *S. aureus*, **(b)** *E. coli*, **(c)** *C. albicans*, and **(d)** *C tropicalis*. The growth control, without ZnNPs, is depicted in blue light. The positive control is shown in red: CIP (for *E coli* and *S. aureus*) and AmB (for *C. albicans* and *C. tropicalis*). Data are presented as the mean ± SD from three independent determinations performed in triplicate. *denotes statistical significance at p < 0.1 for differences between ZnNPs and untreated controls; **p < 0.01 for more significant differences compared with untreated controls. #p < 0.01 differences are considered significant for comparisons of ZnNPs and the antimicrobial controls (CIP and AmB).

As illustrated in **Figure 2**, time-kill curves were constructed to evaluate microbial death overtime at MIC concentrations and above (data not shown). The normal growth curve of each microorganism cultured under untreated conditions, represented by the blue lines, demonstrated a substantial increase in growth over time. In contrast, the growth profiles of ZnNP-treated microorganisms (green lines) showed significant deviations from the untreated controls (**p <0.01). The strongest growth-inhibitory response, indicated by the greatest reduction in viable cells, was observed with ZnNPs treatment.

Specifically, *S. aureus* exhibited the highest rate of cell death, with a marked decline in viability observed after 12 hours (**Figure 2A**, green line, **p < 0.01). In contrast, *E. coli* maintained consistent viability before 24, followed by a significant reduction in viable cells (**Figure 2B**, green line, **p < 0.01). Similarly, the growth profiles of ZnNPs-treated *C. albicans* (**Figure 2C**, green line) and *C. tropicalis* (**Figure 2D**, green line) were significantly altered compared to the untreated controls (**p <0.01). The most substantial inhibitory effect was observed after 12 hours, as indicated by the greatest depletion of viable yeast cells, and more market between 24 and 48 h. Positive controls, represented by the red line in **Figure 2**, included CIP for *E. coli* and *S. aureus*, and AmB for *C. albicans* and *C. tropicalis*. Differences between ZnNPs and the antimicrobial controls (CIP and AmB) are shown in each panel (^#^p<0.01).

### 3.3 Total microbial biofilm biomass susceptibility to ZnNPs

**Figure 3:**
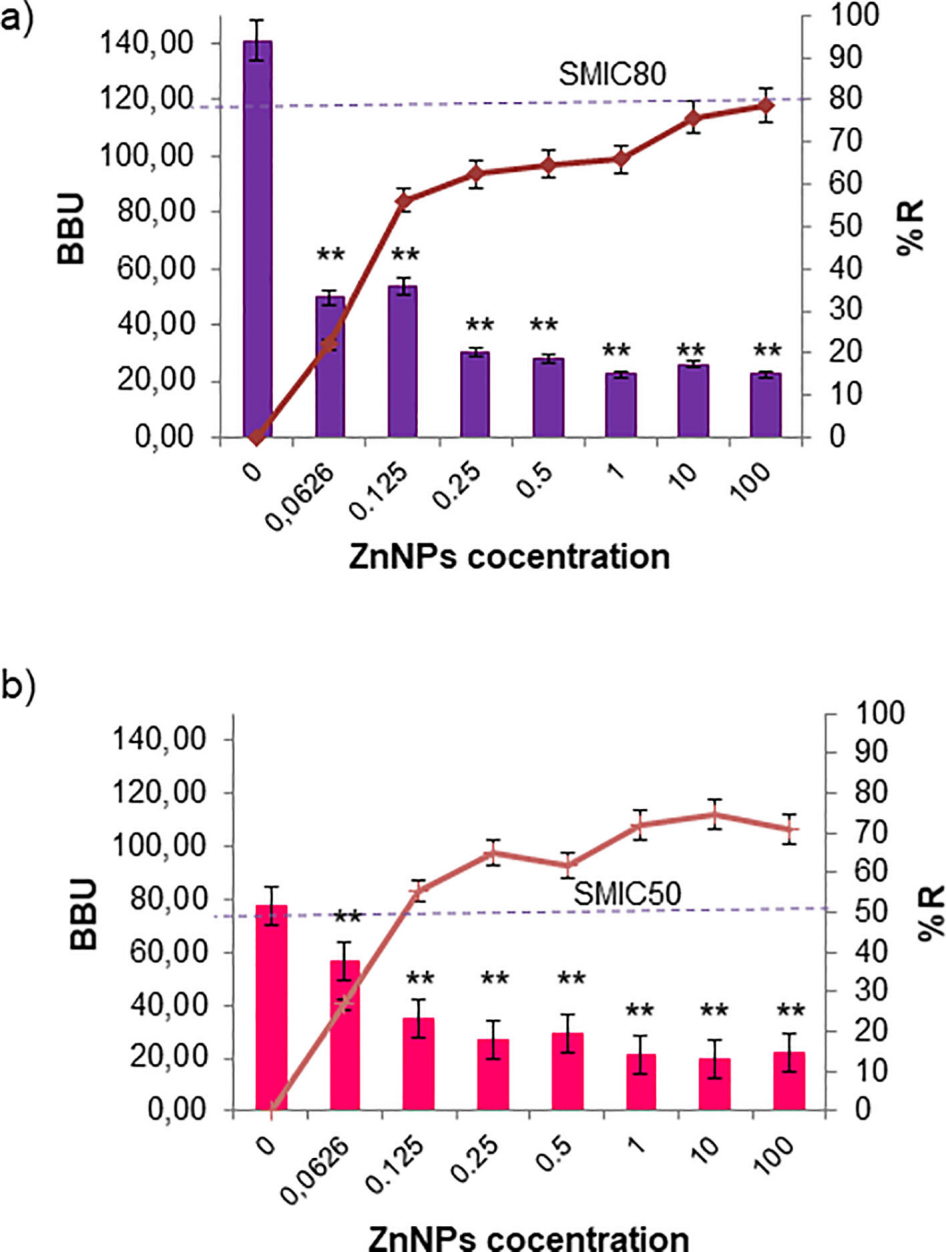
Sessile minimum inhibitory concentration (SMIC50 and SMIC80) for ZnNPs on total bacterial biofilms. **(a)** Mature biofilms of *S. aureus* and **(b)** *E. coli* were exposed to serial dilutions of ZnNPs at concentrations above and below the MIC. CV staining was used to quantify biofilm biomass units (BBUs, bars) and the percentage of biofilm reduction (%R, dotted lines). SMIC50 and SMIC80 are shown for each condition, indicating 50% and 80% reductions, respectively. All experiments were performed in triplicate, with three independent replicates, and the numerical data are presented as means ± standard deviation. Statistical significance is denoted by **p < 0.01 for differences compared to untreated biofilms.

Biofilm formation is a pathogenic mechanism employed by bacteria and fungi, contributing to the complications associated with infections caused by these pathogens. Total mature biofilms of both bacteria and yeasts were subjected to serial dilutions at upper and lower MIC levels. In **Figure 3**, the bar graph represents the total mature biofilm biomass (including sessile cells and extracellular matrix) quantified by crystal violet (CV) staining after antibiofilm activity assays, while the overlaid lines indicate the corresponding percentage of reduction (%R) relative to the untreated biofilms. The results demonstrated that ZnNPs effectively disrupted established mature biofilms of all tested pathogenic strains. *S. aureus* was identified as the strongest biofilm producer, showing greater susceptibility to the antibiofilm effects of ZnNPs compared to untreated controls (**p < 0.01). The SMIC50 value was determined to be 200 µg/mL, which was equal to both the MIC and MBC values. The SMIC80 value was achieved at 5 times this value (**Figure 3A**). Less antibiofilm activity was observed for *E. coli*, where the SMIC50 value was equivalent to the MIC value, however, the SMIC80 value was not achieved, even at concentrations up to 100 times the MIC (**Figure 3B**, **p < 0.01).

**Figure 4:**
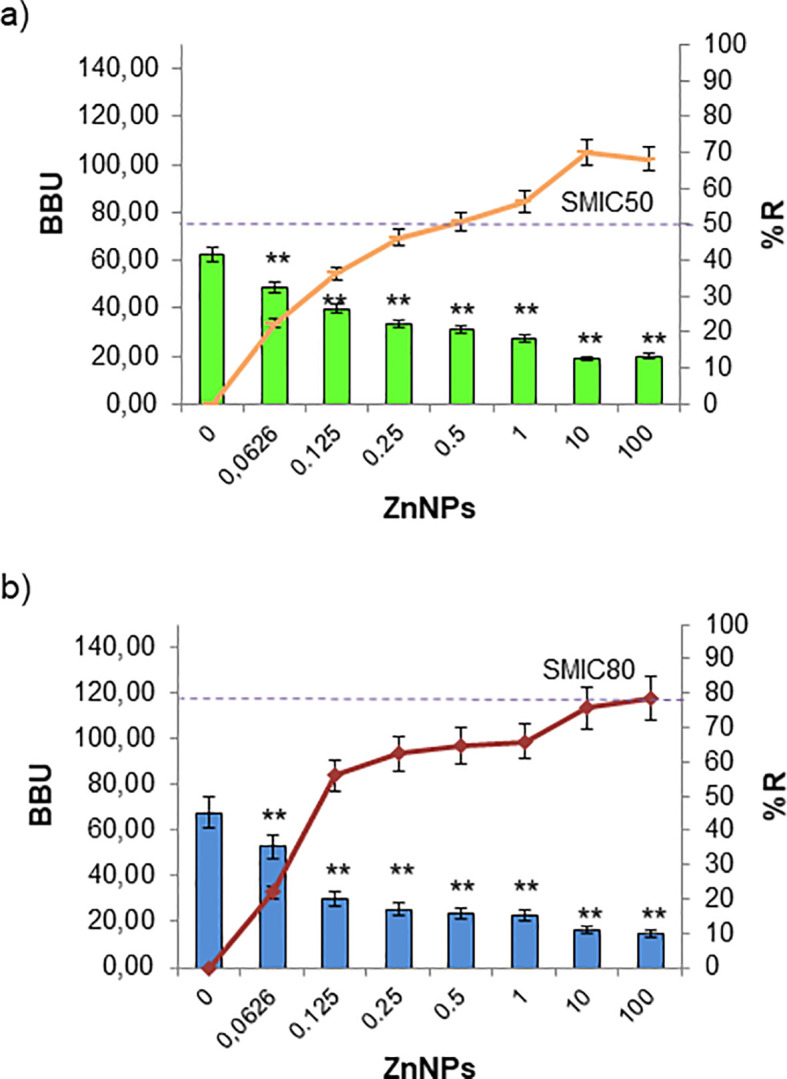
Sessile minimum inhibitory concentration (SMIC50 and SMIC80) for ZnNPs on total fungal biofilms. CV staining was used to quantify biofilm biomass units (BBUs, bars) and the percentage of biofilm reduction (%, dotted lines) for **(a)** *C. albicans* and **(b)** *C. tropicalis* exposed to serial dilutions of ZnNPs at concentrations above and below the MIC. SMIC for 50% and 80% reductions (SMIC50 and SMIC80) in sessile fungal cells are indicated for each strain. All experiments were performed in triplicate, with three independent replicates, and the numerical data are presented as means ± standard deviation. Statistical significance is denoted by **p < 0.01 for differences compared to untreated biofilms.

Regarding the antibiofilm activity of ZnNPs on sessile yeast cells, the SMIC50 value for *C. albicans* was 10 times the MIC (2000 µg/mL). Nevertheless, the SMIC80 threshold was not reached, even when the concentration was increased up to 100-fold above the MIC (**Figure 4A**, **p < 0.01). In the case of *C. tropicalis* exhibited greater sensitivity, achieving the SMIC50 at the same concentration as the MIC (200 µg/mL), indicating higher susceptibility. In contrast, at concentrations 100 times the MIC, the SMIC80 was hardly achieved (**Figure 4B**, **p < 0.01).

**Figure 5:**
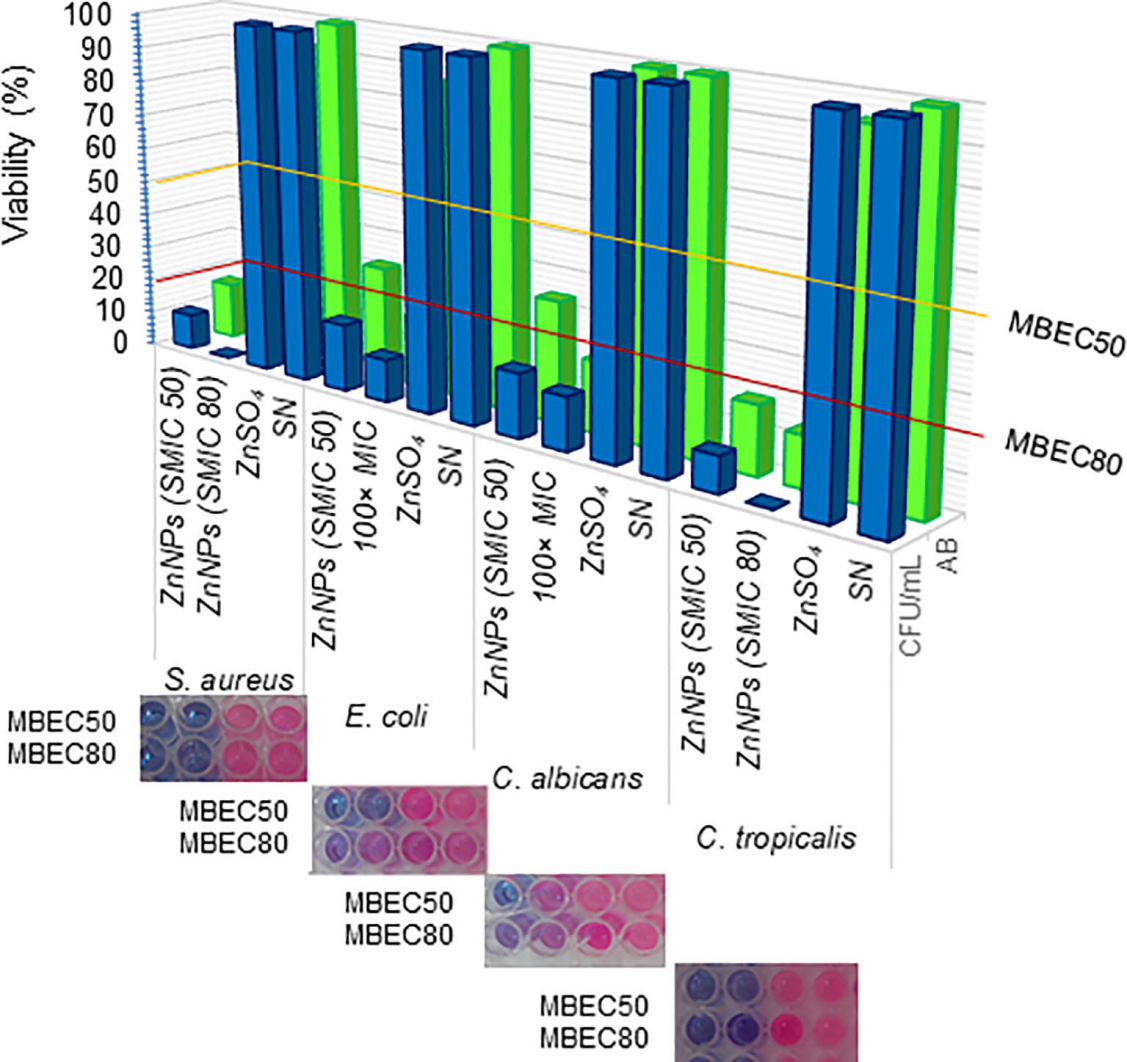
Determination of minimal biofilm eradication concentration (MBEC). Effects of ZnNPs. The metabolic activity of sessile cells was assessed via aerobic respiration following exposure to ZnNPs using resazurin (AB) assays (blue bars and upper panel images). The viability of sessile cells was further quantified through CFU/mL determination by agar plating (green bars). ZnSO_4_ and SN were included as controls. Data are presented as means ± standard deviation (SD from three independent experiments, each performed in triplicate.

Cell viability, assessed through the metabolic activity of microorganisms via aerobic respiration, was determined using resazurin or AB assays. The resazurin method offers a lower detection limit than the OD method, making it well-suited for detecting early metabolic damage induced by ZnNPs. ZnNPs, however, displayed significantly higher microbicidal effects. As shown in **Figure 5**, ZnNPs had an early and significant impact on the metabolic activity of bacterial and yeast cells, resulting in a lower percentage of cell viability across all studied strains. MBEC50 values were achieved for all tested strains. CFU determination via agar plating, widely regarded as the gold standard for biofilm quantification, proved to be more sensitive in our analysis, further confirming reduced cell viability. Additionally, MBEC80 values were successfully achieved for all strains, highlighting the robust efficacy of ZnNPs. In contrast, neither ZnSO_4_ nor SN exhibited microbicidal activity, demonstrating that the metal precursor alone lacked antimicrobial efficacy against the tested organisms. Supplementary material 4 evaluates the synergistic effect of ZnNPs in combination with CIP against *S. aureus* and *E. coli*, as well as with AmB against *C. albicans* and *C. tropicalis*. The percentage of viable cells (% viability) was determined by enumerating colony-forming units and resazurin-resorufin (AB) assay (**Supplementary Figure S4**).

### 3.4 Cellular stress metabolites and OSR in mature biofilms incubated with ZnNPs

**Figure 6:**
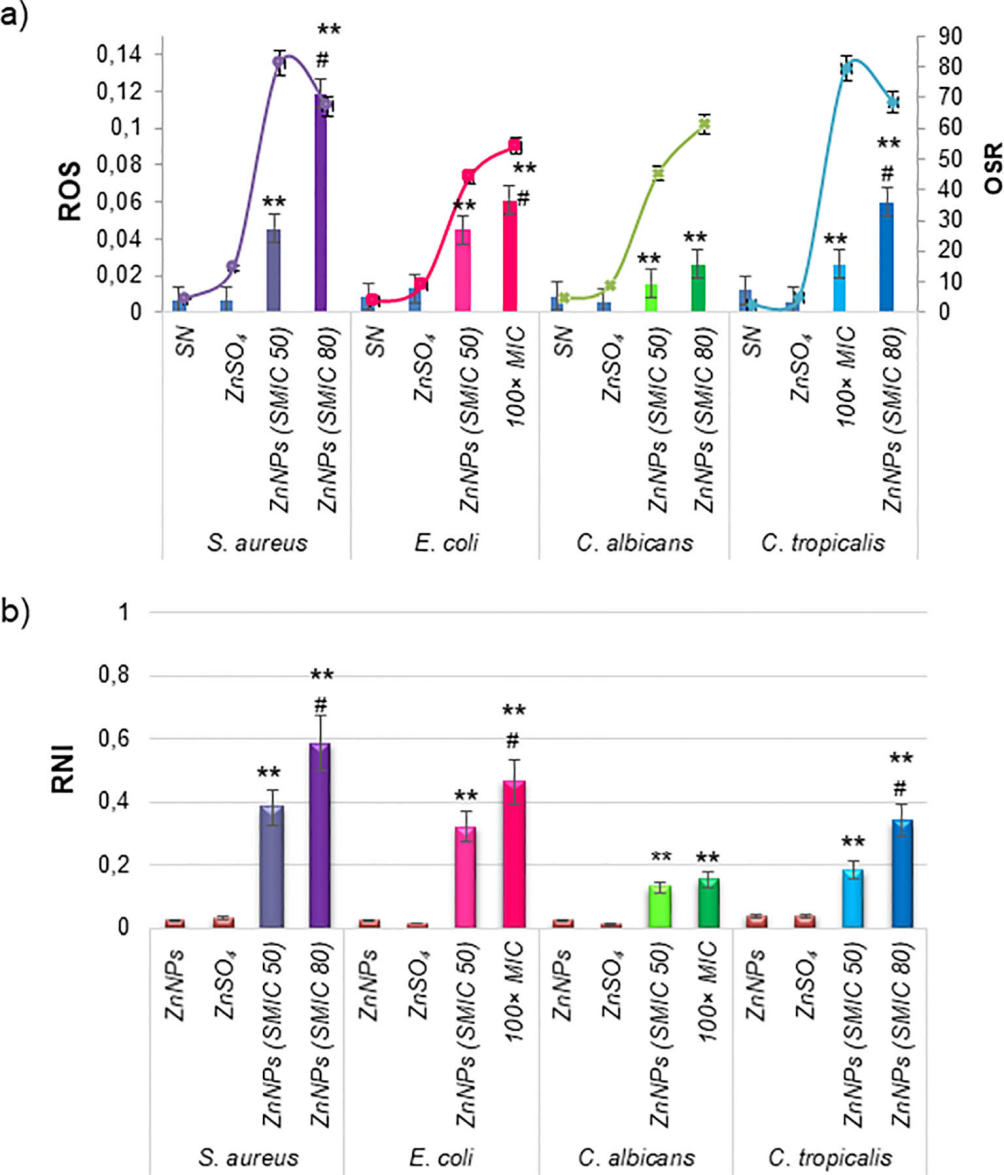
Cellular stress metabolites and OSR in total mature biofilms incubated with ZnNPs. **(a)** Levels of ROS (bars) and OSR results from the FRAP assay (lines). **(b)** Generation of RNI. ZnSO_4_ and SN were included as controls and did not exhibit cellular stress activity. All experiments were conducted in triplicate across three independent experiments, with numerical data presented as means ± standard deviation. Statistical significance is denoted by **p < 0.01 for differences compared to untreated biofilms. Additionally, #p < 0.01 between ZnNPs at SMIC50 and SMIC80.

Oxidative stress was investigated as a potential mechanism of action for ZnNPs on mature biofilms. The production of oxidative metabolites, including ROS and RNI, as well as the OSR (measured via the FRAP assay), were evaluated following treatment with ZnNPs at SMIC50, SMIC80, and 100× MIC levels, as was explained above. ZnNPs treatment resulted in a significant increase in ROS, RNI, and OSR enzymatic activity at SMIC50 levels. **Figure 6A** shows that ROS levels increased in a concentration-dependent manner compared to untreated biofilms (*p < 0.01). Additionally, a differential effect was observed, with higher ROS production at SMIC80 or 100× MIC levels compared to SMIC50 levels across the four strains tested (^#^p < 0.01). RNI generation (**Figure 6B**) showed the greatest accumulation at SMIC80 or 100× MIC levels compared to untreated controls (*p < 0.01), these levels exceeding those of ROS relative to their respective untreated levels. The enzymatic and non-enzymatic antioxidant components were analyzed as indicators of the biofilm’s protective mechanisms against oxidative stress. FRAP levels increased following ZnNPs treatment, with the highest increments observed at SMIC50 and comparatively lower levels at SMIC80, possibly due to increased OSR. Treatment resulted in statistically significant differences compared to untreated biofilms. Overall, these results suggest that biofilm growth was predominantly affected by the accumulation of ROS and RNI. *S. aureus* exhibited the highest ROS and RNI levels. The oxidative stress response was more pronounced at SMIC80, indicating a differential stimulation of sessile cells under ZnNPs treatment.

## 4 Discussion

In recent years, pathogens’ increasing persistence and resistance to antimicrobial agents have underscored the urgent need to develop novel antimicrobial agents and innovative strategies (Vitiello et al., 2023; WHO, 2024). Studies estimate that biofilms contribute to approximately 65% of microbial infections and are associated with 78.2% of human chronic infections (Malone et al., 2017; Paraje, 2023; Coenye et al., 2024). As a result, the scientific community has increasingly prioritized the development of antibiofilm strategies. In this context, traditional antimicrobial drugs are widely recognized as having limited efficacy against biofilms, primarily due to intrinsic and extrinsic resistance mechanisms that exacerbate treatment challenges (Sharma et al., 2023; Coenye et al., 2024). These limitations have driven the exploration of innovative approaches, particularly in biomedical science, with nanotechnology-based solutions garnering significant interest.

Numerous studies have highlighted the significant potential of metal and metal oxide nanoparticles in combating microbial resistance. NPs such as gold (Au), silver (Ag), and titanium (Ti) have demonstrated remarkable efficacy against planktonic cells (Crespo et al., 2016; Da Silva et al., 2022, 2024; Arora et al., 2024; Yakoup et al., 2024). However, their application in eradicating sessile cells within biofilms remains underexplored, emphasizing the need for further research to harness their full potential in addressing biofilm-associated infections effectively. In this regard, ZnNPs have received comparatively less attention, underscoring the need for deeper investigation into their antibiofilm properties. One notable advantage of ZnNPs is that they have been generally recognized as safe by the U.S. Food and Drug Administration (Yousef and Danial, 2012). Studies in the literature mainly address the effects of ZnNPs obtained through chemical synthesis (Applerot et al., 2012; Dwivedi et al., 2014; Lee et al., 2014; Kadiyala et al., 2018; Jasim et al., 2020; Bianchini Fulindi et al., 2023) and plant biological synthesis (Agarwal et al., 2018; Lahiri et al., 2022; Velsankar et al., 2022). Those regarding the effects of ZnNPs obtained through microorganism biosynthesis are more scarce, in particular about ZnNPs obtained with *P. aeruginosa* (Barsainya and Pratap Singh, 2018; Abdo et al., 2021). Thus, limited studies have performed comprehensive evaluations of their antimicrobial activity against both planktonic and sessile cells, or across pathogenic bacteria and fungi. This is especially relevant for green-synthesized ZnNPs, which present a promising avenue for addressing biofilm-associated infections.

The disk diffusion method was employed to evaluate the antimicrobial activity of ZnNPs by measuring the diameters of the inhibition zones, which reflect the antimicrobial susceptibility of the tested microorganisms (Kourmouli et al., 2018; Ihsan et al., 2023). In this sense, by agar diffusion technique demonstrated high efficacy against pathogenic Gram-positive bacteria and Gram-negative bacteria (Hossain, 2024). Their activity has also been reported against fungal phytopathogens such as *Rhizoctonia solani*, *Fusarium* spp., and *Penicillium* spp (Barsainya and Pratap Singh, 2018). However, no further studies have evaluated the sensitivity of planktonic versus sessile biofilm cells to ZnNPs. In this study, we demonstrate that biogenic ZnNPs exhibit potent antimicrobial effects against Gram-positive and Gram-negative bacteria, as well as yeasts, in both planktonic and biofilm forms. Additionally, the inhibition zone data were used to calculate the PIDG values (Himratul-Aznita et al., 2011). A positive PIDG value indicates that the antimicrobial activity is stronger than that of the positive control. Although ZnNPs demonstrated significant antibacterial activity, their PIDG values were negative when compared to the positive controls. Among the tested microorganisms, *S. aureus* exhibited the highest PIDG values, indicating the greatest antimicrobial activity, followed by *E. coli*, *C. tropicalis*, and *C. albicans*, which displayed the lowest activity relative to their respective positive controls. According to other authors, Gram-negative bacteria exhibit greater resistance to ZnNPs because the lipopolysaccharides in their cell walls hinder ZnNP adhesion and internalization (Yu et al., 2014; Abdo et al., 2021).

The broth microdilution method was also utilized to assess the antibacterial activity of biogenic Zn NPs, with MIC/MBC and MIC/MFC results confirming their bactericidal and fungicidal properties. The time-kill kinetics assay quantitatively assesses the antimicrobial activity of an agent over time, focusing on its effects across different microbial growth phases (Chen et al., 2022; Yakoup et al., 2024). This method can determine the bactericidal or fungicidal activity of an agent, defined by a reduction exceeding 3 log_10_ in CFUs, equivalent to a 99.9% eradication of the initial inoculum. The results confirmed the “cidal” effect of ZnNPs, demonstrating significant antimicrobial activity against both bacterial and fungal strains within 48 hours, with significant deviations compared to untreated controls (**p < 0.01). A particularly strong effect was observed on *S. aureus* during both the exponential and stationary growth phases (**p < 0.01). In contrast, *E. coli* showed effects during the stationary phase, particularly before 24 hours. For both *Candida* species, the antimicrobial action occurred during the exponential phase, with marked depletion observed between 24 and 48 hours (**p < 0.01).

Similar to our findings, previously reported studies have demonstrated that ZnNPs effectively inhibit the growth of *S. aureus* planktonic cells, inducing morphological alterations, shape distortions, and a reduction in enterotoxin A, an important virulence factor (El-Masry et al., 2022). Others have emphasized the attachment of ZnO particles to bacterial membranes and the subsequent toxic effects. Specifically, ZnO^-1^, which has a greater surface area, offered a higher availability of zinc atoms, further enhancing bacterial toxicity. The adhesion of ZnO to bacterial membranes induced physical damage, as revealed by research on *S. aureus* and *P. aeruginosa* (Ann et al., 2014). These findings highlight the impact of ZnNPs on cell wall morphology and integrity, suggesting their potential to disrupt normal cellular structure and, consequently, their functionality. Beyond direct contact, ZnNPs also exhibit antibacterial activity via numerous mechanisms like the membrane disruption, DNA damage, enzyme inactivation, and mitochondrial damage, as broadly discussed in this Review (Huq et al., 2023). Earlier research indicates that the interactions with membrane proteins play a significant role in bacterial inactivation, rather than direct physical interactions alone (Zhang et al., 2010).

Other studies have evaluated the antimicrobial efficacy of AgNPs, ZnONPs, and TiO_2_NPs against *Salmonella typhimurium*, *Brucella abortus*, and *C. albicans*, suggesting that nanoparticle solutions exhibit both fungicidal and bactericidal effects on the tested microorganisms (Kareem et al., 2021). Ahmadpour Kermani et al. further demonstrated that TiO_2_NPs and ZnONPs possess antifungal activity against five pathogenic *Candida* species, inhibiting the growth of all tested strains. However, consistent with our results, their antifungal properties were significantly less effective compared to AmB (Ahmadpour Kermani et al., 2021). Abdo et al. reported that the biogenic ZnONPs were concentration-dependent, consistent with our findings. They demonstrated high efficacy against pathogenic Gram-positive bacteria (*S. aureus* and *Bacillus subtilis*), Gram-negative bacteria (*E. coli* and *P. aeruginosa*), and unicellular fungi (*C. albicans*). However, they documented relatively smaller inhibition zones for both Gram-positive and Gram-negative bacteria, as well as fungi (Abdo et al., 2021). In contrast, our results indicate a larger halo of inhibition, suggesting enhanced antimicrobial efficacy against planktonic cells. Moreover, a recent report highlighted that eco-friendly synthesized ZnONPs exhibit greater antimicrobial efficacy against Gram-positive bacteria (*Micrococcus luteus*, *S. aureus*, *Bacillus subtilis*) compared to Gram-negative bacteria (*Agrobacterium tumefaciens*, *Salmonella setubal*, *Enterobacter aerogenes*). These authors emphasize the necessity for further research to elucidate the precise mechanisms underlying the action of these NPs and their broader applications in the medical field (Noor et al., 2024). Our study contributes significantly to this growing body of knowledge by addressing these aspects and providing valuable insights into the antimicrobial properties of green-synthesized ZnNPs and their potential use in medical applications. However, we recognize some limitations, particularly considering that these are *in vitro* studies. For future toxicity studies in human cellular lines, we are considering the inclusion of additional controls.

In contrast to planktonic bacteria, biofilms offer a significant survival advantage to microbial communities. These biofilms are complex microbial communities embedded in a 3D extracellular matrix, primarily composed of polysaccharides, with lesser amounts of proteins, extracellular DNA, lipids, and cellular debris (Paraje, 2023; Sharma et al., 2023; Coenye et al., 2024). They are characterized by strong adhesive capacity and persistent cell population (Da Silva et al., 2021, 2024). Biofilms is a major challenge in modern medicine, as most existing antimicrobial agents are designed to target free-floating microbial cells (Zhao et al., 2023). Currently, there is no standard method for evaluating the efficacy of new antibiofilm drugs (Hossain, 2024). In this study, we utilized a combination of methodologies—the CV assay, resazurin-based method, and CFU plate counting—to evaluate the antibiofilm activity of biogenic ZnNPs on mature biofilms. The CV assay’s main limitation is its measurement of total biofilm biomass (BBU), as it stains all components of the biofilm, including live and dead bacteria, as well as the extracellular matrix. Nonetheless, this assay is unable to identify viable bacteria as distinct from non-viable ones (Sharma et al., 2023; Coenye et al., 2024). Viability and vitality assays are critical for assessing the effectiveness of novel therapeutic approaches, with stain-based methods offering speed and objectivity. To address the limitations of the CV assay, we employed two additional cell viability assays. The first was a resazurin-based method, which evaluates cellular metabolic activity by assessing the redox status of microbial cells (Herman et al., 2024). The second method was CFU counting, which is considered the gold standard for determining viable sessile cells by disrupting the total mature biofilm through sonication (Da Silva et al., 2021, 2022, 2024). The combination of these three methodologies provides a broader perspective on the antibiofilm effects of ZnNPs, enabling a more comprehensive assessment of their antimicrobial action. By integrating biofilm biomass measurements and viability assays, we demonstrated that ZnNPs exert significant effects on biofilms formed by both bacteria and fungi. Additionally, we were able to determine important parameters to characterize their action, specifically the SMIC and the MBEC. While the MIC is an important parameter for planktonic cells, the determination of the SMIC is particularly relevant for agents that can inhibit sessile cells, which may be used to design effective antibiofilm therapies. Sessile cells constitute only 5–30% of the volume in mature biofilms, with the remaining volume consisting of the extracellular matrix (Sharma et al., 2023; Coenye et al., 2024). The results obtained in this study, as evaluated by the CV assay, suggest that the extracellular matrix present in the biofilm significantly disrupts the total biofilm when treated with ZnNPs. In addition to determining the SMIC at 50% and 80%, we assessed the MBEC for mature biofilms using both resazurin-based staining and CFU counts. These results reflect the number of viable cells remaining after treatment and demonstrate the effective eradication of mature biofilm formation by various microbial species.

Another important aspect is that by different methods it was confirmed that ZnSO_4_ and SN did not exhibit inhibition zones or microbicidal activity, indicating that the metal precursor alone lacked antimicrobial efficacy against the tested organisms. In contrast, the biogenic ZnNPs displayed significantly higher microbicidal effects. Different reports have been postulated that the biomolecules stabilizing the NPs and forming their corona also play a role in their antimicrobial effects (Nandhini et al., 2024). These corona components can interact with the microbial cell membrane, followed by internalization and contributing to the control of biofilm-forming microbial pathogens (Kundu et al., 2014; Sass et al., 2018). In addition, siderophores can chelate iron and other metals essential for microbial survival. If siderophores form part of the corona, metal chelation may further aid in biofilm control. In this sense, it has been reported that iron denial produced by pyoverdine was the main mechanism by which pyoverdine inhibited *A. fumigatus* biofilm (Roberts et al., 2024). *Pseudomonas aeruginosa* was selected for this studies due to its well-documented ability to biosynthesize various metallic nanoparticles. This bacterium employs versatile metabolic pathways and secretes biomolecules such as reductase enzymes, proteins, and extracellular polysaccharides, which not only facilitate the bioreduction of metal ions but also serve as natural capping and stabilizing agents for the nanoparticles. These biological molecules adsorbed on the nanoparticle surface give rise to what is known as the nanoparticle corona—a dynamic layer of biomolecules that plays a crucial role in determining nanoparticle characteristics such as colloidal stability, surface charge, aggregation behavior, and biological interactions. In the case of biosynthesized nanoparticles, the corona often includes varying proportions of carbohydrates, proteins, and aliphatic compounds, which can influence their antimicrobial properties, biocompatibility, and functional behavior in environmental and biomedical applications (Alamdari et al., 2022; Ali et al., 2024).

Most research has focused on the antimicrobial properties of ZnNPs, primarily attributed to the generation of ROS, which is the most widely studied and accepted mechanism underlying the antibacterial activity of ZnO. ROS include highly reactive ionic species and free radicals, such as superoxide anion, hydroperoxyl radical, hydrogen peroxide, and hydroxyl radical. These species can damage DNA, lipids, proteins, and cell membranes, ultimately triggering cell death (Yousef and Danial, 2012; Peralta et al., 2018). However, limited studies have explored the effects of RNI and the resulting imbalance on bacteria and yeast (Arce Miranda et al., 2011; Baronetti et al., 2011; Angel Villegas et al., 2015; Da Silva et al., 2021; Quinteros et al., 2021a). In this study, we determined the toxicity mechanism of ZnNPs, demonstrating potent antibacterial, antifungal, and antibiofilm activities mediated by the generation of both ROS and RNI. The results indicate a disturbance in the prooxidant/antioxidant balance, favoring the overproduction of ROS and RNI. Biofilms treated with ZnNPs displayed an ability to respond to the stress generated during treatment when exposed to the SMIC50. However, at the SMIC80, this imbalance was more pronounced due to the excessive production of free radicals and an insufficient OSR. Higher levels of ROS and RNS accumulation inside of biofilm were observed, resulting in a significant oxidative imbalance.

The RNI, particularly nitric oxide (NO), were identified as the main contributors to the prooxidant/antioxidant balance within biofilms (Sirelkhatim et al., 2015). Due to its small size and neutral charge, NO is able to diffuse freely through the channels and voids of mature biofilms (Arce Miranda et al., 2011; Da Silva et al., 2022, 2024). Inside the biofilm, NO reacts with metals and other radicals to generate additional RNI that can interact with various biofilm components, including the outer cell wall-embedded proteins, which are among the primary targets (Mirhosseini et al., 2019; Costa-Orlandi et al., 2021; Oliveira et al., 2023). This process was concentration-dependent and the accumulation of RNI within biofilms can destabilize the matrix by interacting with polysaccharides and eDNA, resulting in oxidative damage, such as DNA cleavage. Previously, we reported that NO is an effective antibiofilm agent in *S. aureus* with the ability to disperse biofilm-associated microorganisms, penetrate the matrix, and disrupt biofilm structure (Arce Miranda et al., 2011; Angel Villegas et al., 2015; Da Silva et al., 2021). *In vitro* studies have shown c-di-GMP is understood to be regulated by endogenously produced NO during the late stages of biofilm development as a means to induce biofilm dispersal. This NO-mediated dispersal mechanism is conserved across various species, including *P. aeruginosa*, *S. aureus*, *E. coli*, *Fusobacterium nucleatum*, and *Vibrio cholerae* (Altammar, 2023; Burlec et al., 2023; Oliveira et al., 2023; Piluk et al., 2024; Nguyen et al., 2025).

At present, researchers are increasingly focusing on NPs composed of metals and metal oxides as innovative antimicrobial agents. These NPs possess unique physical and chemical properties, including small size, high surface area-to-volume ratio, distinctive surface chemistry, and the ability to effectively penetrate biofilms (Ann et al., 2014; Arora et al., 2024; Yakoup et al., 2024). ZnNPs, in particular, have received significant attention as a promising class of antibacterial agents. This interest arises from their remarkable physicochemical properties and enhanced surface area (Kadiyala et al., 2018; Noor et al., 2024). Zinc, an essential trace element in the metabolism of plants, humans, and animals, plays a critical role in human physiology. It also contributes to ecological sustainability, owing to its solubility, low toxicity, and biodegradability (Król et al., 2017; Kalaba et al., 2024). Moreover, zinc can promote biofilm dispersal by disrupting cellular balance. In addition to these advantages, ZnNPs are cost-effective and commercially viable, making them suitable for a wide range of applications. Their unique properties position them as highly favorable candidates for both industrial and biomedical purposes (Zhang et al., 2010; Sirelkhatim et al., 2015; Mirhosseini et al., 2019; Costa-Orlandi et al., 2021; Altammar, 2023; Burlec et al., 2023; Oliveira et al., 2023; Noor et al., 2024; Piluk et al., 2024; Nguyen et al., 2025). Despite the promising properties of these ZnNPs, some limitations of this study should be noted. *In vitro* models do not consider the environmental factors of the host and other biotic signals occurring *in vivo* (Lebeaux et al., 2013), that may influence both the stability and the activity of the nanoparticle (Mba and Nweze, 2021).

## 5 Conclusion

This study demonstrates the antibacterial, antifungal, and antibiofilm activity of biologically synthesized ZnNPs from *P. aeruginosa*, emphasizing their effectiveness against both planktonic and sessile forms of the tested microorganisms. Our findings represent the first comprehensive analysis of the multifunctional antimicrobial properties of these eco-friendly ZnNPs, including microbial toxicity mechanisms triggered by the induction of ROS and RNI, with a disturbance in the prooxidant/antioxidant balance. This research contributes to a deeper understanding of their potential in managing biofilm-associated infections. The clinical relevance of ZnNPs is highlighted by their potential to inform effective strategies for eradicating biofilms, particularly in light of the increasing prevalence of biofilm-related complications among at-risk patients. Their properties render ZnNPs highly valuable for treating infections. This suggests their versatility for various applications in the biomedical field, including the development of new antimicrobial agents, with implications for healthcare, agriculture, and various industries.

## Data Availability

The datasets presented in this article are not readily available. Requests to access the datasets should be directed to gabrielaparaje@gmail.com.

## Supplementary material

Supplementary Figure 2 ZnNPs biosynthesis and characterization. **(a)** UV–vis spectra of biosynthesized ZnNPs by *P. aeruginosa* culture. **(b)** Zeta potential of the biosynthesized ZnNPs. **(c)** Representative TEM image of the biosynthesized ZnNPs. **(c)** Representative SEM images of ZnNPs.

Supplementary Figure 3 Characterization and analysis of capping. **(a)** FT-IR spectrum of ZnNPs biosynthesized by P*. aeruginosa*. **(b)** SDS-PAGE silver staining, line 1: supernatant; line 2: ZnNPs sample. The arrows show protein bands. **(c)** Fluorescence spectrum of ZnNPs.

Supplementary Figure 4 Study of synergistic antimicrobial activity. Synergistic effect of ZnNPs in combination with CIP against *S. aureus* and *E. coli*, as well as with Amb against *C. albicans* and *C. tropicalis*. The percentage of viable cells (% viability) was determined using colony-forming units per mL (CFU.mL^-1^, grey bars) and resazurin (AB) assays (blue bars). The numerical data correspond to the relative values (treated/untreated ratio). All experiments were conducted in triplicate across three independent experiments, with numerical data presented as means ± standard deviation. Statistical significance is indicated as follows: **p* < 0.01 for differences compared to untreated biofilms and # *p* < 0.01 for significant differences between treatments with antibiotics (CIP) or antifungal (Amb) and ZnNPs.
