# Supplementary figures and images for: Antibacterial, antifungal, and antibiofilm activities of biogenic zinc nanoparticles against pathogenic microorganisms
Source: Front Cell Infect Microbiol. 2025 Jul 14;15:1545119. doi: 10.3389/fcimb.2025.1545119 (PMC12301384; doi:10.3389/fcimb.2025.1545119)

## Supplementary Material 1

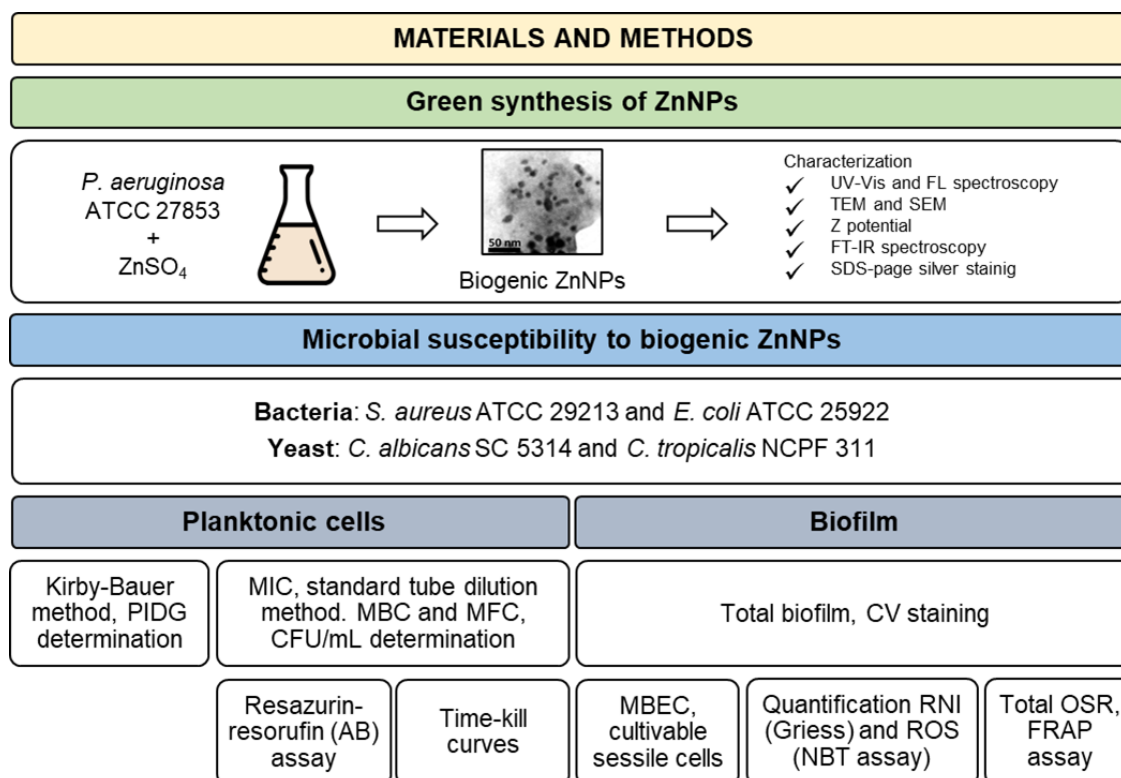

**Figure S1. Schematic summary of materials and methods.**

Supplement: Supplementary file 1 [file DataSheet1.pdf]

a)

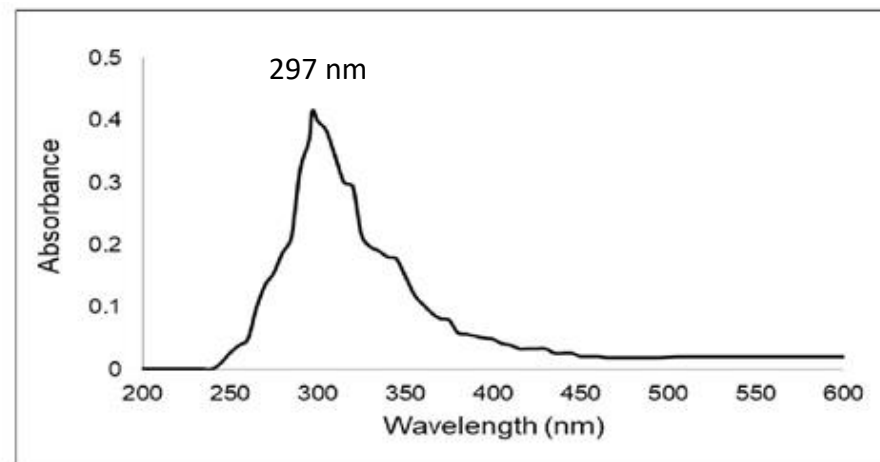

b)

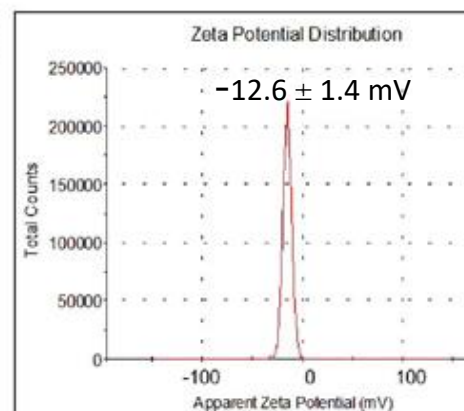

c)

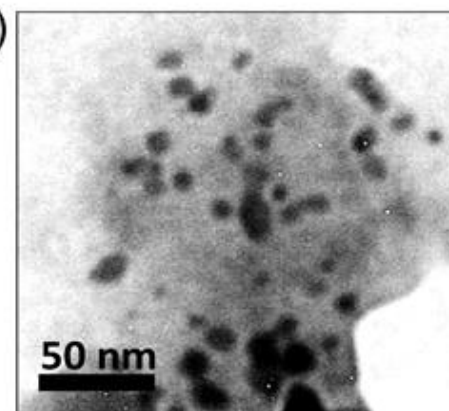

d)

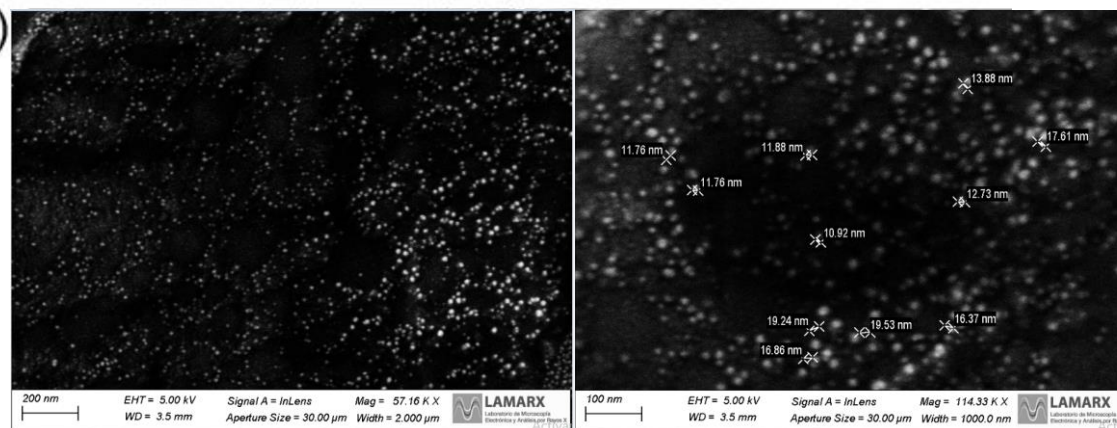

Supplement: Supplementary Figure 2 — ZnNPs biosynthesis and characterization. (a) UV–vis spectra of biosynthesized ZnNPs by P. aeruginosa culture. (b) Zeta potential of the biosynthesized ZnNPs. (c) Representative TEM image of the biosynthesized ZnNPs. (c) Representative SEM images of ZnNPs. [file DataSheet2.pdf]

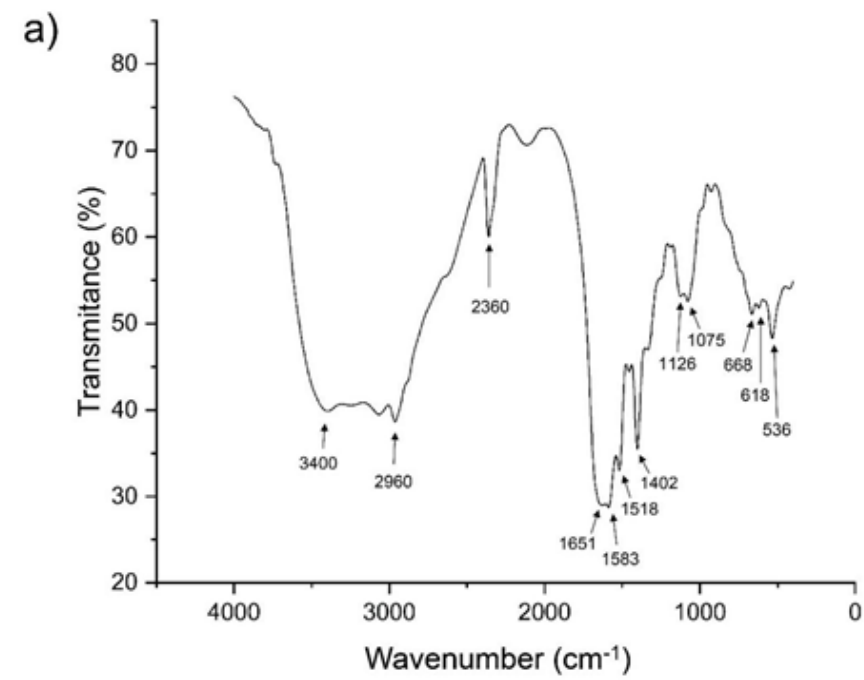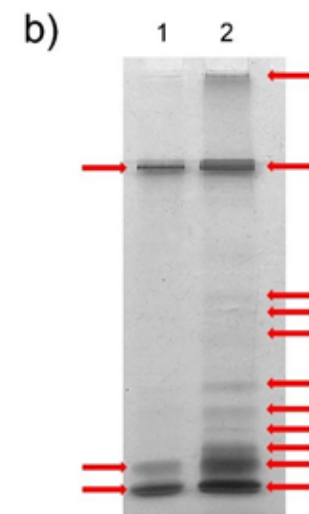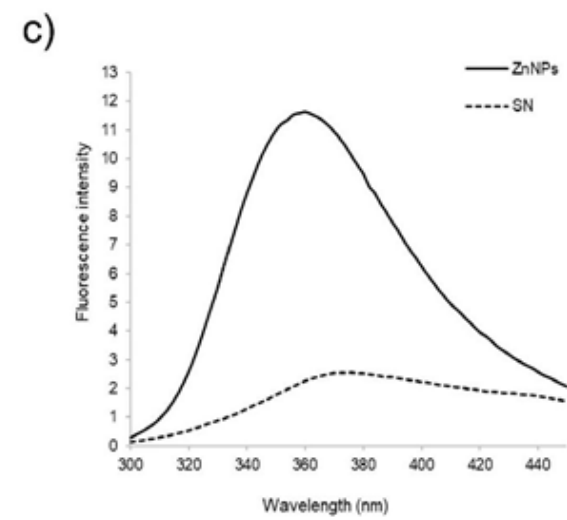

Supplement: Supplementary Figure 3 — Characterization and analysis of capping. (a) FT-IR spectrum of ZnNPs biosynthesized by P. aeruginosa. (b) SDS-PAGE silver staining, line 1: supernatant; line 2: ZnNPs sample. The arrows show protein bands. (c) Fluorescence spectrum of ZnNPs. [file DataSheet3.pdf]

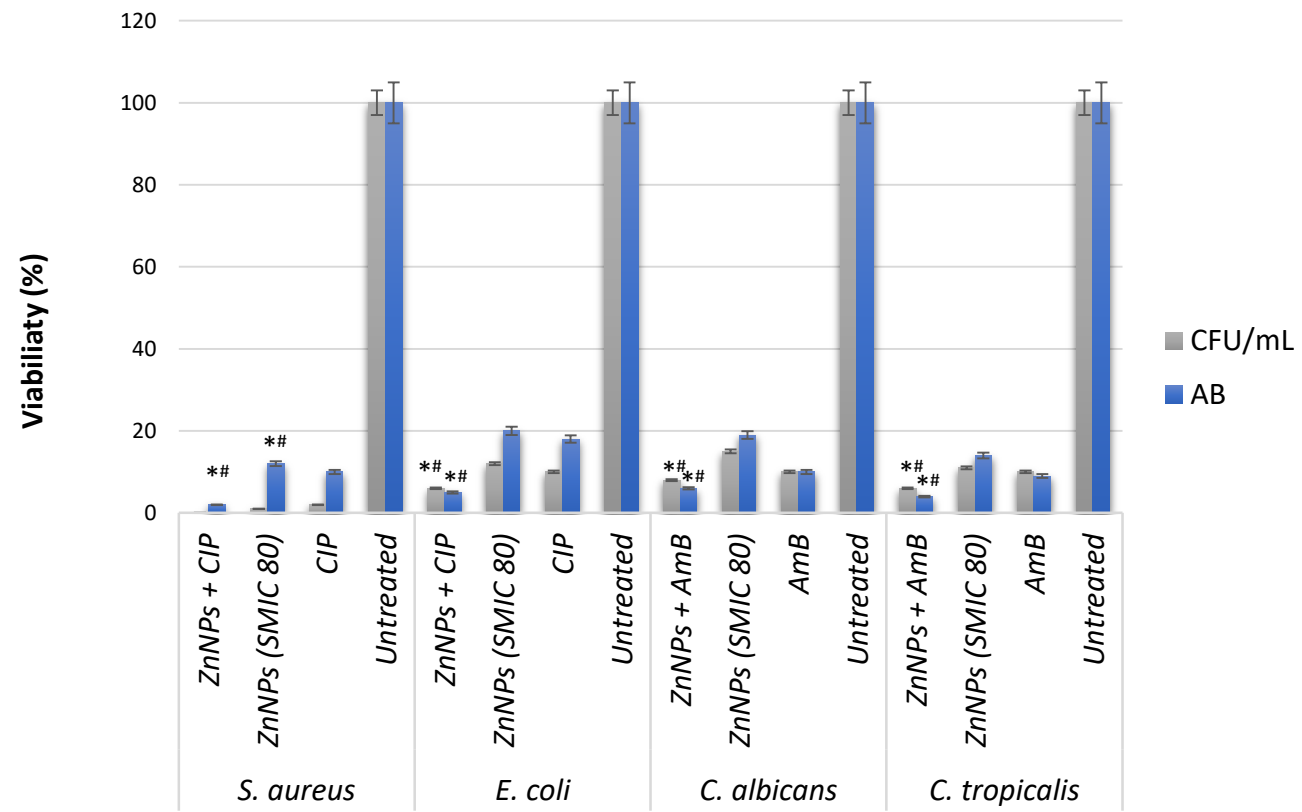

Supplement: Supplementary Figure 4 — Study of synergistic antimicrobial activity. Synergistic effect of ZnNPs in combination with CIP against S. aureus and E. coli, as well as with Amb against C. albicans and C. tropicalis. The percentage of viable cells (% viability) was determined using colony-forming units per mL (CFU.mL-1, grey bars) and resazurin (AB) assays (blue bars). The numerical data correspond to the relative values (treated/untreated ratio). All experiments were conducted in triplicate across three independent experiments, with numerical data presented as means ± standard deviation. Statistical significance is indicated as follows: *p < 0.01 for differences compared to untreated biofilms and # p < 0.01 for significant differences between treatments with antibiotics (CIP) or antifungal (Amb) and ZnNPs. [file DataSheet4.pdf]
